# In Search of More Leaps to Realize the Precision Medicine of Migraine

**DOI:** 10.3390/ijms242417375

**Published:** 2023-12-12

**Authors:** Mamoru Shibata

**Affiliations:** Department of Neurology, Tokyo Dental College Ichikawa General Hospital, Chiba 272-8513, Japan; mshibata@tdc.ac.jp

Migraine, clinically characterized by recurrent headache attacks of moderate-to-severe intensity, is the second most disabling disease in terms of years lived with disability [1]. As a physician–scientist, I should emphasize that migraine is not just a headache. Migraine headache is a manifestation of a hyperactivated trigeminal pain system. In addition, most migraine sufferers complain of photophobia, phonophobia, and osmophobia in the ictal period [2]. Some patients suffer from allodynia, which may occur in extracephalic locations [3]. Taken together, the clinical picture of migraine attacks can be interpreted as an uncontrollable state of multiple sensory modalities [4]. Pain induces negative emotions and autonomic reactions, which aggravates the agony of migraine attacks. The presence of aura adds another layer of throes. Scintillating scotoma, the most common symptom of migraine aura, not only impairs visual function but also causes severe anxiety and even terror. Migraine aura is known to be caused by cortical spreading depolarization (CSD), a wave of abrupt, near-complete breakdown of neuronal transmembrane ion gradients in the cerebral cortex accompanied by dynamic changes in cerebral blood flow [5]. This is a clear indication that migraine pathophysiology involves the central nervous system (CNS). Moreover, people with migraine, not infrequently, report appetite changes and fatigability in the phase commencing up to 48 h before the onset of pain in migraine without aura (MO) and before the aura in migraine with aura (MA), which is called the prodromal/premonitory phase [6,7]. Functional imaging data point to the role of the hypothalamus in the emergence of prodromal/premonitory symptoms [8,9]. Clearly, multiple neural system perturbations amalgamate to generate the complex symptomatology of migraine.

Familial hemiplegic migraine (FHM) is known to be caused by genetic mutations in *CACNA1A*, *ATP1A2*, and *SCN1A*, all of which lead to enhanced neuronal excitability [10]. Meanwhile, ordinary migraine is considered a polygenic disorder [11]. Recent genome-wide association studies have identified more than 120 risk loci for developing migraine [12]. The results show that neuronal and vascular abnormalities may contribute to migraine pathogenesis, which is congruent with the tenet that migraine is a very complex disorder. Intriguingly, evidence is emerging that MO and MA have distinct genetic backgrounds [13]. It is envisioned that genetic analysis for each phenotype of migraine will clarify the molecular mechanism underlying its complex symptomatology.

There have been numerous reports on the alterations in brain structure and network in patients with migraine, although it remains unclear whether each of these abnormalities is a cause or a consequence of the disease state associated with migraine [14]. Aberrant sensory signal processing has been observed in a manner dependent on the phase. Intriguingly, the trigeminal nociceptive signal processing pattern is shown to exhibit a lack of habituation during the interictal phase [15,16], and the trigeminal nociceptive transmission becomes increasing enhanced toward the subsequent ictal phase [17]. These dynamic functional changes likely lead to the development of the cyclicity of migraine attacks.

Where does the migraine headache occur? The trigeminal afferents innervating the dura mater are regarded as important loci of migraine headache generation [18,19]. The dural meninges, especially around the sinuses, are enriched in mast cells and lymphocytes and serve as a neuroimmune interface [20]. Moreover, within the trigeminal ganglion (TG) tissue, satellite glial cells, which surround TG neurons, are known to actively produce immune mediators and nitric oxide [21]. However, the precise role of inflammation in migraine pathogenesis remains undetermined. Calcitonin gene-related peptide (CGRP), a 37-amino acid neuropeptide derived from a *CALCA* splice variant, is released from trigeminal afferents and TG neurons, which somehow results in the activation of the trigeminal system [22,23]. The importance of this molecule in migraine pathogenesis is highlighted by the therapeutic success of CGRP blockade in treating and preventing migraine attacks [24]. Surprisingly, the inhibition of only one molecule provides a significant protective effect against the complex disorder. Whether the blockade of other neuropeptides, such as pituitary adenylate cyclase-activating polypeptide (PACAP), amylin, and adrenomedullin, exerts an antimigraine effect is a matter of great interest among researchers [25]. The activation of nociceptive TG neurons conveys pain signals to the trigeminal nucleus caudalis and then to other brainstem nuclei, the hypothalamus, the basal ganglia, and the thalamus [26]. This bottom-up signal transmission-associated activation and basal CNS dysfunction should play a pivotal role in the complex clinical manifestations of migraine.

To decipher the complexity of migraine pathophysiology, more research is required in the form of system biology. This Special Issue entitled “Molecular and Cellular Neurobiology of Migraine 1” is a collection of noteworthy research contributions that provide important insight to this end.
